# Supplementary material for: Tracking COVID-19 using online search
Source: NPJ Digit Med. 2021 Feb 8;4:17. doi: 10.1038/s41746-021-00384-w (PMC7870878; doi:10.1038/s41746-021-00384-w)
Supplement: Supplementary file 1 — Reporting Summary [file 41746_2021_384_MOESM1_ESM.pdf]

nature research

Corresponding author(s): Vasileios Lampos

Last updated by author(s): 2020/12/18

Reporting Summary

Nature Research wishes to improve the reproducibility of the work that we publish. This form provides structure for consistency and transparency in reporting. For further information on Nature Research policies, see our Editorial Policies and the Editorial Policy Checklist.

Statistics

For all statistical analyses, confirm that the following items are present in the figure legend, table legend, main text, or Methods section.

n/a	Confirmed	
☒	☐	The exact sample size (n) for each experimental group/condition, given as a discrete number and unit of measurement
☐	☒	A statement on whether measurements were taken from distinct samples or whether the same sample was measured repeatedly
☐	☒	The statistical test(s) used AND whether they are one- or two-sided<br>*Only common tests should be described solely by name; describe more complex techniques in the Methods section.*
☐	☒	A description of all covariates tested
☒	☐	A description of any assumptions or corrections, such as tests of normality and adjustment for multiple comparisons
☐	☒	A full description of the statistical parameters including central tendency (e.g. means) or other basic estimates (e.g. regression coefficient) AND variation (e.g. standard deviation) or associated estimates of uncertainty (e.g. confidence intervals)
☒	☐	For null hypothesis testing, the test statistic (e.g. F, t, r) with confidence intervals, effect sizes, degrees of freedom and P value noted<br>*Give P values as exact values whenever suitable.*
☒	☐	For Bayesian analysis, information on the choice of priors and Markov chain Monte Carlo settings
☒	☐	For hierarchical and complex designs, identification of the appropriate level for tests and full reporting of outcomes
☐	☒	Estimates of effect sizes (e.g. Cohen's d, Pearson's r), indicating how they were calculated

Our web collection on statistics for biologists contains articles on many of the points above.

Software and code

Policy information about availability of computer code

Data collection	Online search data was obtained using Python from an Application Interface (API) developed by Google and known as the Google Health Trends API. Access to this API needs to be authorised by Google after an application process that is open to all research groups.
Data analysis	We have used MATLAB version 2019a and standard functions or packages for the data analysis. Our Code Availability section paragraph provides further details.

For manuscripts utilizing custom algorithms or software that are central to the research but not yet described in published literature, software must be made available to editors and reviewers. We strongly encourage code deposition in a community repository (e.g. GitHub). See the Nature Research guidelines for submitting code & software for further information.

Data

Policy information about availability of data

All manuscripts must include a data availability statement. This statement should provide the following information, where applicable:

- Accession codes, unique identifiers, or web links for publicly available datasets
- A list of figures that have associated raw data
- A description of any restrictions on data availability

The online search and the swabbing scheme data sets that support the findings of this study are available from Google and the Royal College of General Practitioners (RCGP) or Public Health England (PHE), respectively. Restrictions apply to the availability of these data sets, which were used under license for the current study, and so are not publicly available. These data sets are however available from the authors upon reasonable request and with the respective permission of Google and RCGP/PHE. The rest of the data sets that this study is based on are available at https://figshare.com/projects/Tracking_COVID-19_using_online_search/81548.

Field-specific reporting

Please select the one below that is the best fit for your research. If you are not sure, read the appropriate sections before making your selection.

☐ Life sciences ☒ Behavioural & social sciences ☐ Ecological, evolutionary & environmental sciences

For a reference copy of the document with all sections, see nature.com/documents/nr-reporting-summary-flat.pdf

Behavioural & social sciences study design

All studies must disclose on these points even when the disclosure is negative.

Study description	Models for COVID-19 prevalence using aggregate statistics about online searches
Research sample	The analysis is based on a sample of user search sessions at the Google search engine in 8 countries (US, UK, Australia, Canada, France, Italy, Greece, and South Africa). This sample is subsequently sub-sampled by Google to ensure user privacy. It is estimated that approximately a sample of about 10-15% of the actual searches is retained and used to estimate search term frequencies. We only have access to these final aggregate search term frequencies.
Sampling strategy	NA - The sub-sampling strategy (10-15% of the entire set of searches) is conducted by Google and the exact approach is not known to us. We use the data obtained from the Google Health Trends API as is. Our understanding is that this sub-sampling is essential in order to retain user privacy by removing content (online search queries) that could potentially reveal a user's identity.
Data collection	NA - The data sets used in this study were provided to us. We did not generate a novel data set.
Timing	September 30, 2011 to May 24, 2020
Data exclusions	NA
Non-participation	NA
Randomization	NA

Reporting for specific materials, systems and methods

We require information from authors about some types of materials, experimental systems and methods used in many studies. Here, indicate whether each material, system or method listed is relevant to your study. If you are not sure if a list item applies to your research, read the appropriate section before selecting a response.

Materials & experimental systems

n/a	Involved in the study	
☒	☐	Antibodies
☒	☐	Eukaryotic cell lines
☒	☐	Palaeontology and archaeology
☒	☐	Animals and other organisms
☒	☐	Human research participants
☒	☐	Clinical data
☒	☐	Dual use research of concern

Methods

n/a	Involved in the study	
☒	☐	ChIP-seq
☒	☐	Flow cytometry
☒	☐	MRI-based neuroimaging
