# Supplementary material for: Tracking COVID-19 using online search
Source: NPJ Digit Med. 2021 Feb 8;4:17. doi: 10.1038/s41746-021-00384-w (PMC7870878; doi:10.1038/s41746-021-00384-w)
Supplement: Supplementary file 2 — Supplementary Information [file 41746_2021_384_MOESM2_ESM.pdf]

Supplementary Information for "*Tracking COVID-19 using online search*"

Vasileios Lampos[1,*], Maimuna S. Majumder[2,3], Elad Yom-Tov[4], Michael Edelstein[5,6], Simon Moura[1], Yohhei Hamada[7], Molebogeng X. Rangaka[7,8], Rachel A. McKendry[9,10], and Ingemar J. Cox[1,11]

[1]Department of Computer Science, University College London, London, UK
[2]Computational Health Informatics Program, Boston Children's Hospital, Boston, MA, USA
[3]Department of Pediatrics, Harvard Medical School, Boston, MA, USA
[4]Microsoft Research, Herzeliya, Israel
[5]National Infection Service, Public Health England, London, UK
[6]Department of Population Health, Faculty of Medicine, Bar-Ilan University, Israel
[7]Institute for Global Health, University College London, London, UK
[8]Division of Epidemiology and Biostatistics, University of Cape Town, Cape Town, South Africa
[9]London Centre for Nanotechnology, University College London, London, UK
[10]Division of Medicine, University College London, London, UK
[11]Department of Computer Science, University of Copenhagen, Copenhagen, Denmark

[*]Corresponding author, v.lampos@ucl.ac.uk

Supplementary Methods

Additional information about the data sets

Confirmed COVID-19 cases and deaths were obtained from the European Centre for Disease Prevention and Control (ECDC) at ecdc.europa.eu/en/publications-data/download-todays-data-geographic-distribution-covid-19-cases-worldwide. All search queries that were considered in our experiments are available online at figshare.com/projects/Tracking_COVID-19_using_online_search/81548. News articles were considered as COVID-19-related, if their title or main text included one of the terms:

- `κορονοϊός', `κορονοϊού', `κορωνοϊός', `κορωνοϊού', `κορωνοϊοί', `κορονοϊοί', 'covid', 'covid-19', 'covid 19', 'covid19', 'coronavirus', and 'ncov' for Greece, or
- 'covid', 'covid-19', 'covid 19', 'covid19', 'coronavirus', 'ncov' for the rest of the countries.

Causal relationship between clinical data for Italy and online searches in other countries

We perform a block-wise Granger causality test[1], as implemented by the function `gctest` in MATLAB®, to assess whether the time series of confirmed cases or deaths in Italy Granger-cause the frequency of search terms in other countries of our analysis. We use a lag of 2 temporal instances, i.e. an autoregressive formulation that uses data up to and including the previous 2 time steps (days). Granger-causality is confirmed when the p-value of the statistical test is less or equal to .05. We focus the analysis on a period from February 17, 2020 to April 19, 2020 (confirmed cases) or April 25, 2020 (deaths) to make it more relevant to the onset of the search increase. The end dates of this analysis are determined by adding a 4-week period after the corresponding peak in confirmed cases or deaths in Italy. The outcome of this causal analysis is that cases and deaths in Italy Granger-caused the frequency of 27.4% (SD: 8.5%) and 27.1% (SD: 9.9%) of the considered search terms on average across the other 7 countries in our analysis. When we weight these rates by the symptom probability of occurrence (Supplementary Table 1), the percentages do not change significantly (26%, SD: 6.1% for cases and 27.6%, SD: 10.9% for deaths).

Additional equations

The min-max normalisation of a vector $\mathbf{x} \in \mathbb{R}^n$ will result to a vector $\hat{\mathbf{x}} \in [0,1]^n$ by performing the following operation:

$$\hat{\mathbf{x}} = \frac{\mathbf{x} - \min(\mathbf{x})}{\max(\mathbf{x}) - \min(\mathbf{x})}\,. \tag{1}$$

The z-score normalisation (or standardisation) of a vector $\mathbf{x} \in \mathbb{R}^n$ will result to a vector $\hat{\mathbf{x}} \in \mathbb{R}^n$ by performing the following operation:

$$\hat{\mathbf{x}} = \frac{\mathbf{x} - \mu(\mathbf{x})}{\sigma(\mathbf{x})}, \tag{2}$$

where $\mu(\cdot)$ and $\sigma(\cdot)$ denote the mean and standard deviation functions, respectively.

The mean absolute error (MAE) between an estimated time series $\hat{\mathbf{y}} \in \mathbb{R}^n$ and the corresponding ground truth $\mathbf{y} \in \mathbb{R}^n$ is equal to

$$\mathrm{MAE}(\hat{\mathbf{y}}, \mathbf{y}) = \frac{1}{n} \sum_{i=1}^{n} |\hat{y}_i - y_i| . \tag{3}$$

A smoothed search query frequency s_i for a day i using a window of the previous D days (including day i) is equal to

$$s_i = \frac{1}{\sum_{p=1}^{D} \frac{1}{p}} \sum_{p=1}^{D} \frac{x_{i-p+1}}{p}, \tag{4}$$

where x_{i-p+1} denotes the raw (non smoothed) frequency for day $i-p+1$.

The Squared Exponential (SE) covariance function (or kernel), commonly used in a Gaussian Process model[2], is defined by

$$k_{\mathrm{SE}}(\mathbf{x}, \mathbf{x}') = \sigma^2 \exp\left(-\frac{(\mathbf{x} - \mathbf{x}')^2}{2\ell^2}\right), \tag{5}$$

where $\mathbf{x}$ and $\mathbf{x}'$ denote rows of an input matrix $\mathbf{X} \in \mathbb{R}^{n \times m}$, ℓ and σ both $\in \mathbb{R}_{>0}$ are the lengthscale and scaling (variance) parameters, respectively.

Supplementary Figures and Tables

Supplementary Figure 1 compares the frequency of search queries that include the words "the", "weather" or "coronavirus" in the United States (US) and the United Kingdom (UK) to illustrate the unprecedented characteristics in the volume of online searches during the COVID-19 pandemic. Supplementary Table 1 shows the probability of specific symptom categories for people who have contracted SARS-CoV-2 based on a survey by the National Health System (NHS) and Public Health England (PHE) in the UK[3]. Supplementary Figure 2 depicts a similar result to Figure 1 (main manuscript), with the difference that the scores are solely based on the first few hundred (FF100) symptom categories[3] without including generic queries about coronavirus. Supplementary Figure 3 is also similar to Figure 1 (main manuscript), but this time the weighting of queries is uniform (equal weights for all symptoms). Supplementary Figure 4 shows a comparison between the unsupervised model with minimised news media effects from Figure 1 (main manuscript) and the corresponding deaths caused by COVID-19; it also shifts back the deaths time series to maximise the correlation between the two signals. Supplementary Figure 5 shows progressive versions of Figure 3 (main manuscript), i.e. all intermediate model outputs (training was conducted on a daily basis). Supplementary Figure 6 compares the estimates from the transfer learning models to the ones from the unsupervised models with minimised news media effects. Supplementary Figure 7 shows additional results from the correlation and regression analysis. Supplementary Figures 8, 9, and Table 2, show the performance results and corresponding plots for the forecasting tasks of deaths and confirmed COVID-19 cases. Supplementary Figure 10 compares the frequency of searches that include search terms about COVID-19-related symptoms and more generic information about COVID-19 that is less likely to be an indicator of an actual infection. Finally, Supplementary Figure 11 shows the average daily news articles proportion about COVID-19 across all countries included in our analysis.

Supplementary References

1. Granger, C. W. J. Investigating causal relations by econometric models and cross-spectral methods. *Econometrica* **37**, 424–438 (1969).
2. Rasmussen, C. E. & Williams, C. K. I. *Gaussian Processes for Machine Learning* (MIT Press, 2006).
3. Boddington, N. L. *et al.* COVID-19 in Great Britain: epidemiological and clinical characteristics of the first few hundred (FF100) cases: a descriptive case series and case control analysis. [Preprint]. *Bull. World Heal. Organ.* doi:10.2471/BLT.20.265603 (2020).

Symptom	Probability
Cough	.777
Fatigue	.709
Fever	.601
Headache	.567
Muscle ache	.509
Appetite loss	.441
Shortness of breath	.404
Sore throat	.386
Joint ache	.339
Runny nose	.325
Loss of the sense of smell	.291
Diarrhoea	.276
Sneezing	.239
Nausea	.236
Vomiting	.087
Altered consciousness	.068
Nose bleed	.060
Rash	.052
Seizure	.008

Supplementary Table 1. Probability of symptom occurrence for people who have contracted SARS-CoV-2 as reported in the FF100 survey conducted by NHS/PHE in the UK.

Country	7 days ahead			14 days ahead		
	AR-F	SAR-F	PER-F	AR-F	SAR-F	PER-F
**US**	6090.02 (4159.88)	5147.06 (4188.73)	5053.74 (4775.49)	11037.60 (8624.17)	6703.70 (7295.66)	5665.03 (5423.37)
**UK**	1672.20 (1235.59)	1046.45 (1035.15)	846.94 (830.41)	2326.82 (1464.47)	1525.97 (1381.28)	1256.80 (1273.08)
**Australia**	123.59 (90.33)	42.62 (42.89)	11.80 (9.78)	217.74 (92.53)	192.63 (114.35)	24.86 (27.30)
**Canada**	392.17 (247.74)	426.98 (358.46)	273.06 (329.19)	717.30 (444.85)	414.10 (341.87)	414.83 (320.46)
**France**	1058.79 (717.59)	824.56 (552.89)	675.14 (851.49)	2050.87 (953.91)	1518.12 (586.42)	1210.69 (1022.56)
**Italy**	1297.69 (508.56)	681.91 (417.17)	604.20 (311.72)	2782.43 (1189.31)	1603.71 (845.22)	1149.54 (506.22)
**Greece**	30.70 (17.05)	28.03 (18.32)	22.37 (32.19)	40.80 (18.46)	25.97 (19.02)	23.37 (27.27)
**South Africa**	374.96 (241.57)	297.92 (211.29)	186.34 (141.94)	448.41 (307.28)	407.52 (253.51)	299.26 (196.18)
**Norm. mean**	0.231 (0.161)	0.164 (0.143)	0.119 (0.143)	0.387 (0.235)	0.271 (0.204)	0.183 (0.172)

Supplementary Table 2. Average mean absolute error and standard deviation (in parentheses) of forecasting models (7 and 14 days ahead) for daily confirmed COVID-19 cases in 8 countries. The last row contains min-max normalised averages across countries, methods, and forecasting tasks to account for the different ranges in different countries. **AR-F**: autoregressive forecasting using past confirmed cases; **SAR-F**: combined online search and autoregressive forecasting; **PER-F**: persistence model.

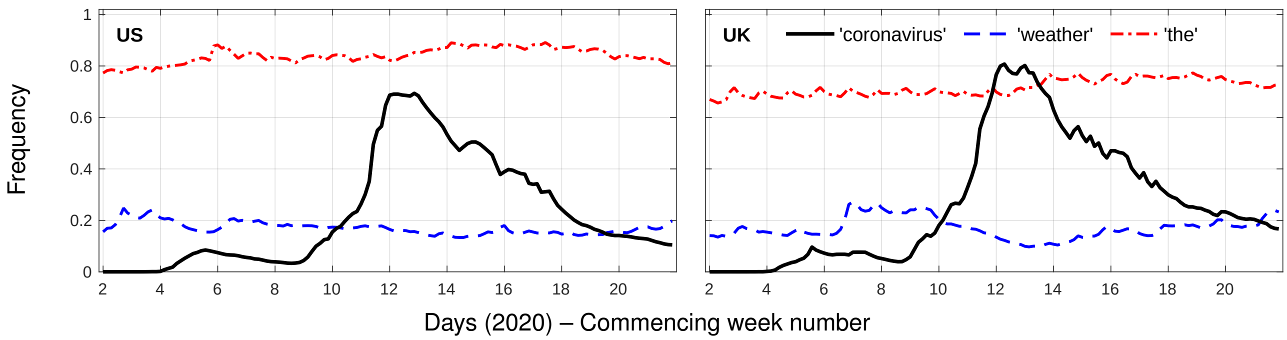

Supplementary Figure 1. Normalised daily frequency time series of all Google search queries that include the keywords "coronavirus", "weather", or "the" in the US and the UK. Time series are smoothed using a 14-day harmonic mean.

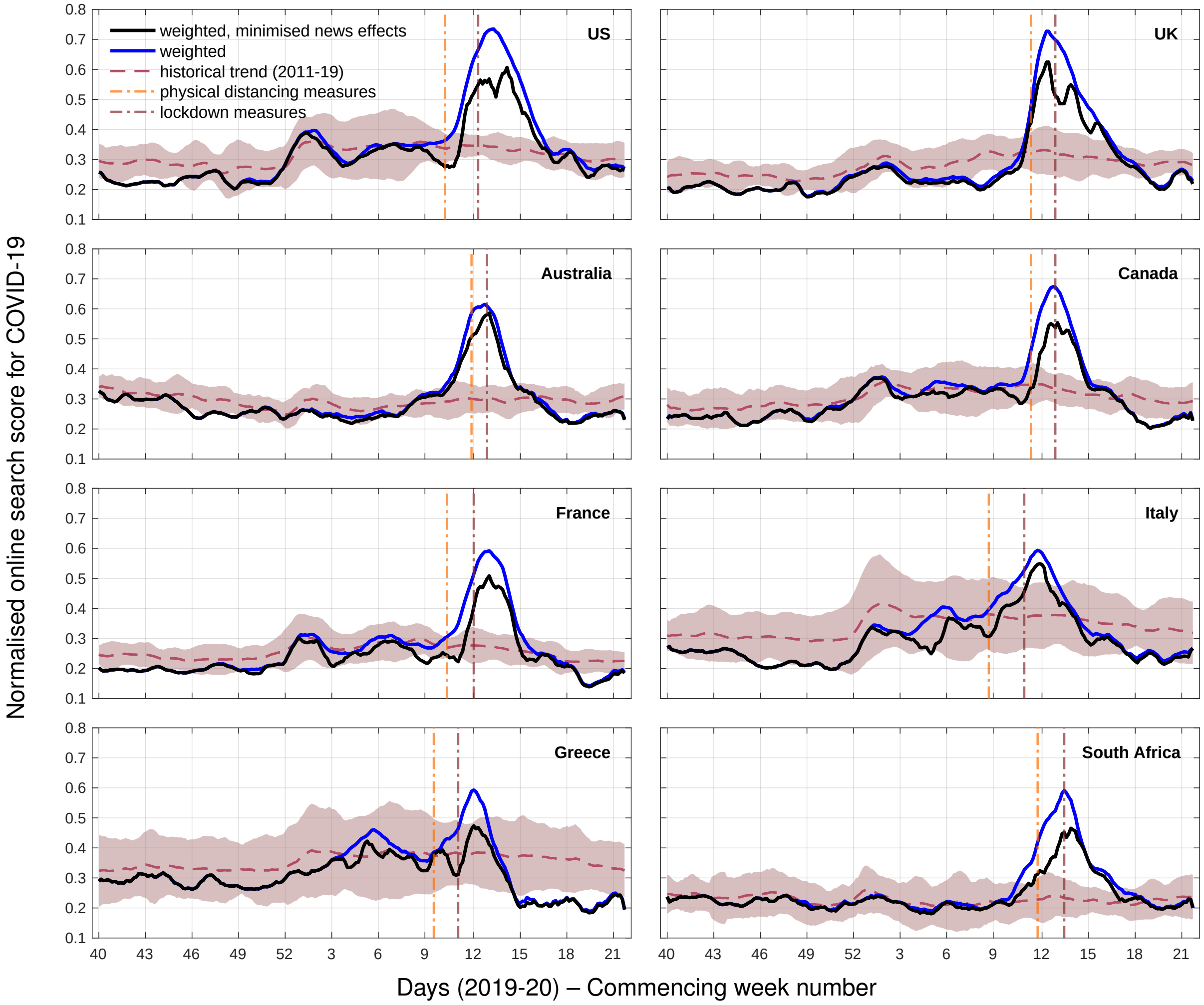

Supplementary Figure 2. Online search based scores for COVID-19-related symptoms as identified by the FF100 survey for 8 countries from September 30, 2019 to May 24, 2020 (all inclusive). Query frequencies are weighted by symptom occurrence probability (blue line) and have news media effects minimised (black line). These scores are compared to an average 8-year trend of the weighted model (dashed line) and its corresponding 95% confidence intervals (shaded area). Application dates for physical distancing or lockdown measures are indicated with dash-dotted vertical lines; for countries that deployed different regional approaches, the first application of such measures is depicted. All time series are smoothed using a 7-point moving average, centred around each day.

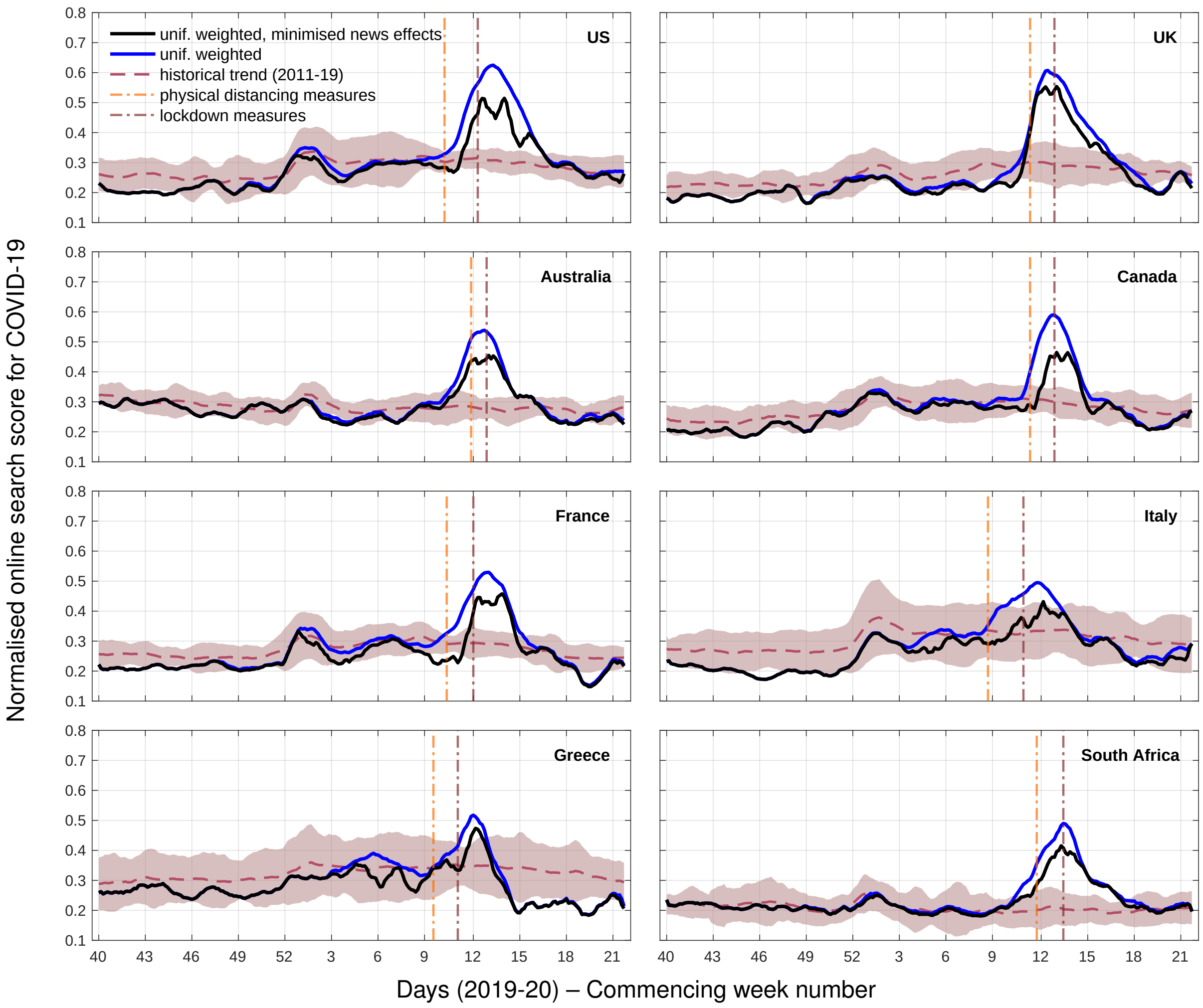

Supplementary Figure 3. Online search scores for COVID-19-related symptoms as identified by the FF100 survey, in addition to queries with coronavirus-related terms, for 8 countries from September 30, 2019 to May 24, 2020 (all inclusive). Query frequencies are uniformly weighted (blue line), and have news media effects minimised (black line). These scores are compared to an average 8-year trend of the uniformly weighted model (dashed line) and its corresponding 95% confidence intervals (shaded area). Application dates for physical distancing or lockdown measures are indicated with dash-dotted vertical lines; for countries that deployed different regional approaches, the first application of such measures is depicted. All time series are smoothed using a 7-point moving average, centred around each day.

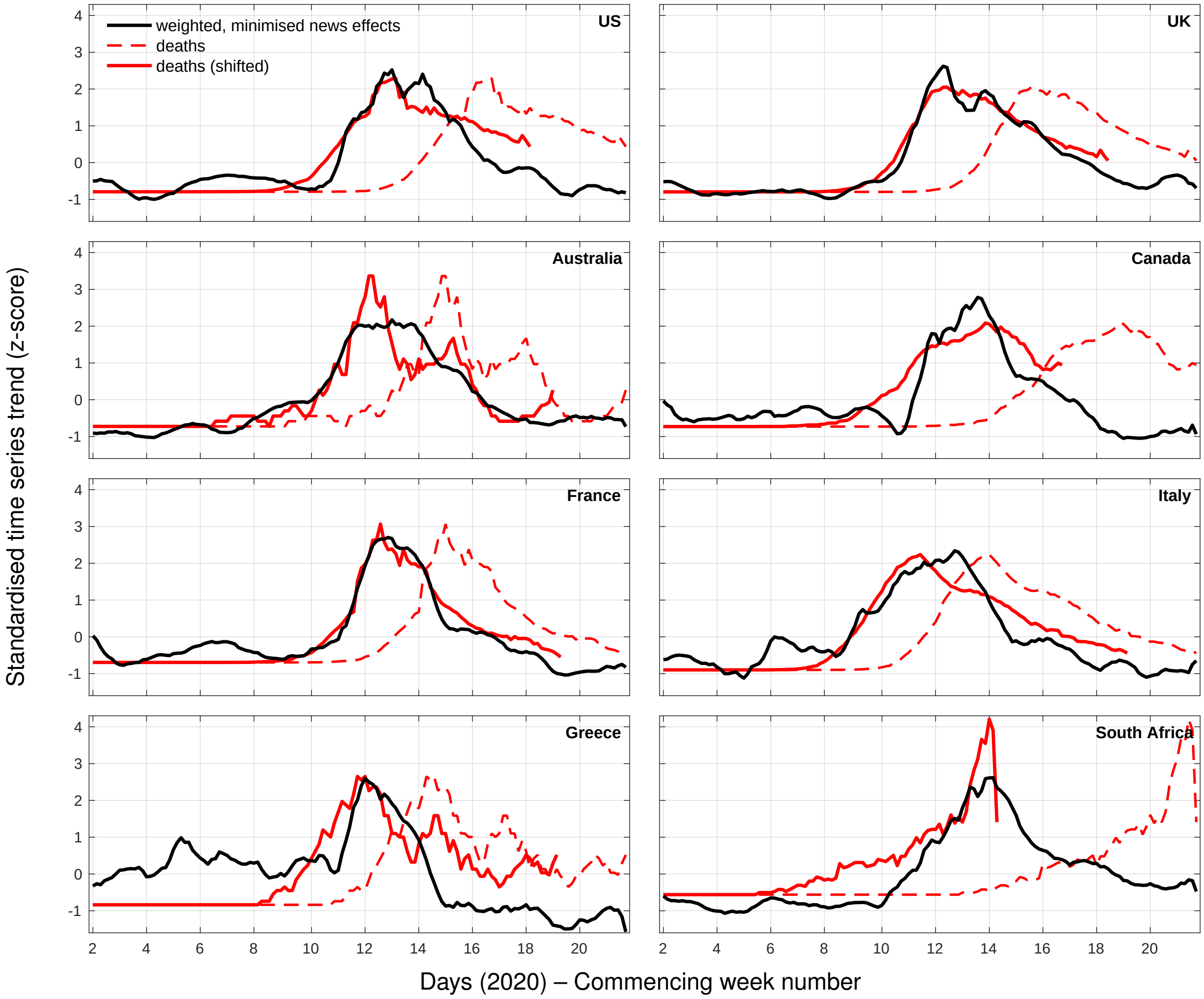

Supplementary Figure 4. Comparison between online search scores with minimised news media effects (black line) with deaths caused by COVID-19 (dashed red line), as well as deaths shifted back (red line) such that their correlation with the online search scores is maximised. The deaths time series are shifted back by a different number of days for each country: 25 days (US), 23 days (UK), 19 days (Australia), 35 days (Canada), 17 days (France), 18 days (Italy), 18 days (Greece), and 52 days (South Africa). All time series are smoothed using a 7-point moving average, centred around each day.

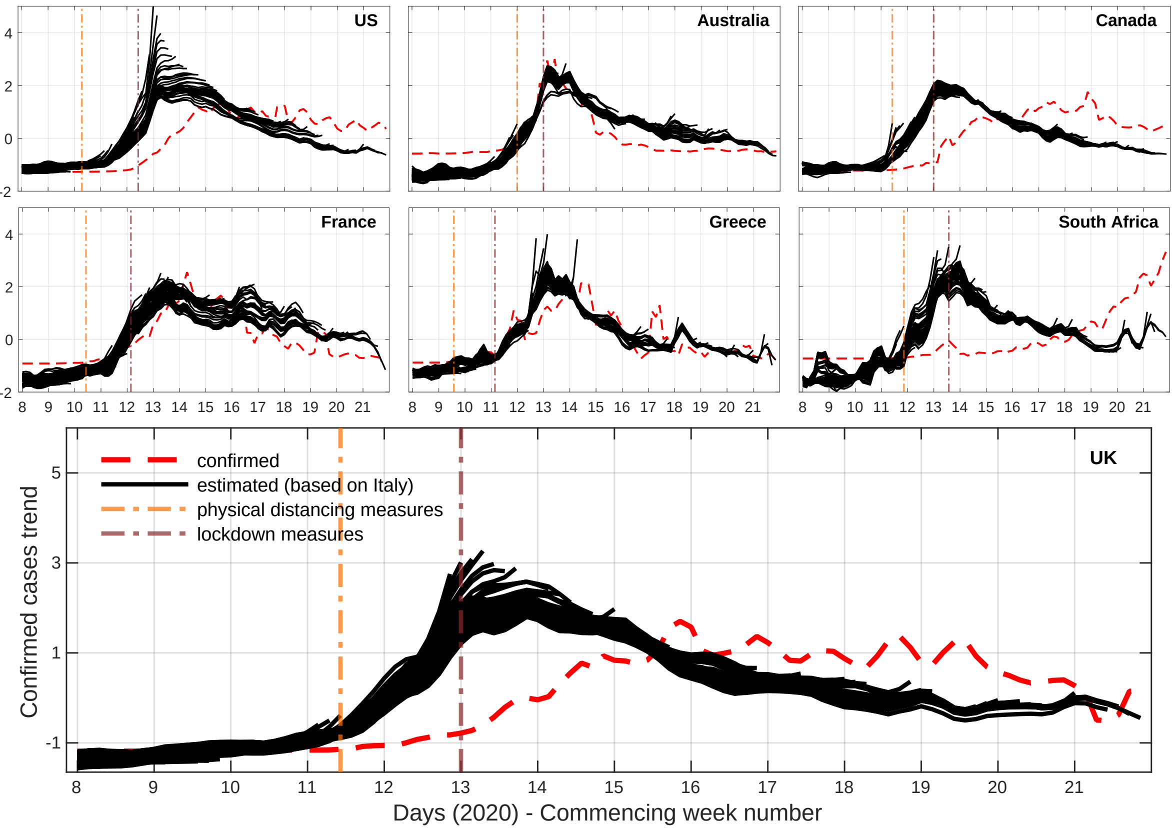

Supplementary Figure 5. Transfer learning models based on online search data for 7 countries and their temporal progression using Italy as the source country. The figures show an ongoing (updated on a daily basis) estimated trend for confirmed COVID-19 cases compared to the reported one. The solid line represents the mean estimate from an ensemble of models. Application dates for physical distancing or lockdown measures are indicated with dash-dotted vertical lines; for countries that deployed different regional approaches, the first application of such measures is depicted. Time series are standardised and smoothed using a 3-point moving average, centred around each day. We use this minimum amount of smoothing to remove some of the noise for visualisation purposes and maintain our ability to compare the transferred models to the corresponding clinical data.

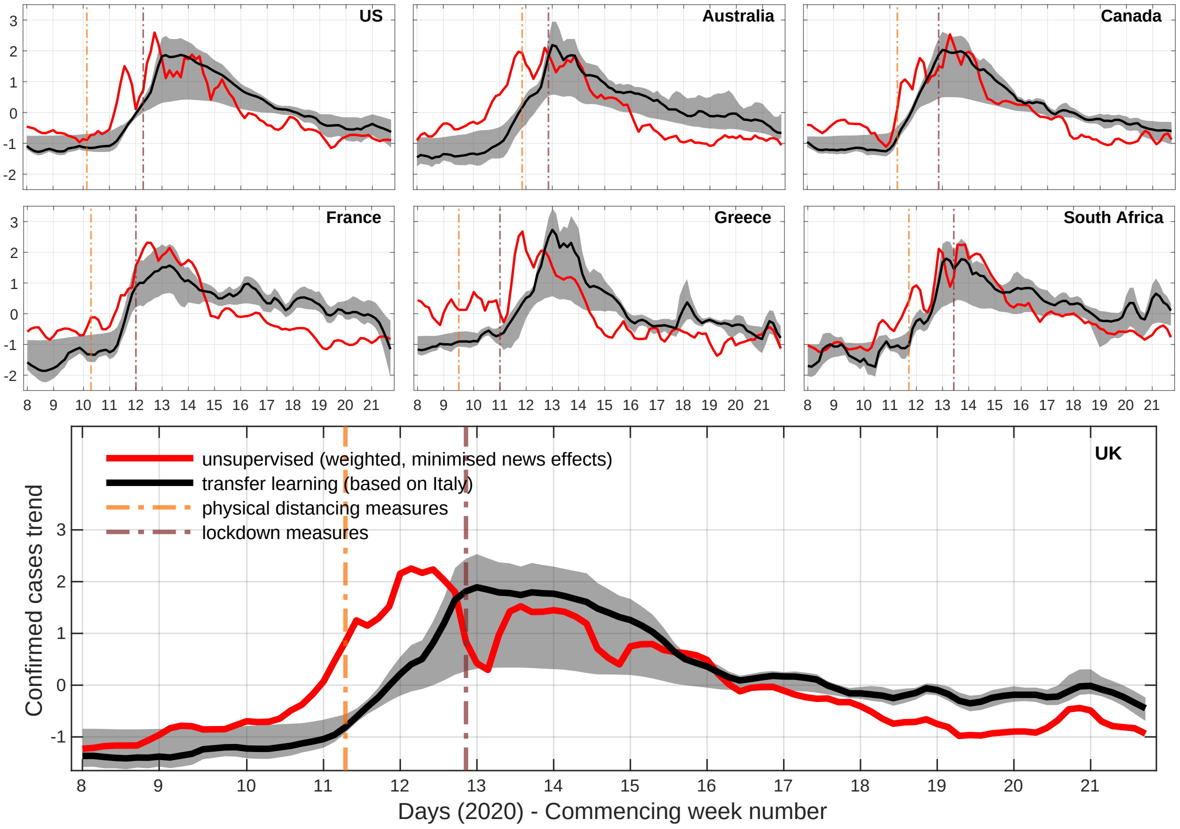

Supplementary Figure 6. Comparison between transfer learning and unsupervised (weighted, minimised news effects) models based on online search data for 7 countries. Italy is the source country for the transfer learning models. Both time series are standardised to allow comparison. The solid black line represents the mean estimate from an ensemble of transferred models. The shaded area shows 95% confidence intervals based on all transferred model estimates. The solid red line shows the estimates from the unsupervised model. Application dates for physical distancing or lockdown measures are indicated with dash-dotted vertical lines; for countries that deployed different regional approaches, the first application of such measures is depicted. Time series are smoothed using a 3-point moving average, centred around each day.

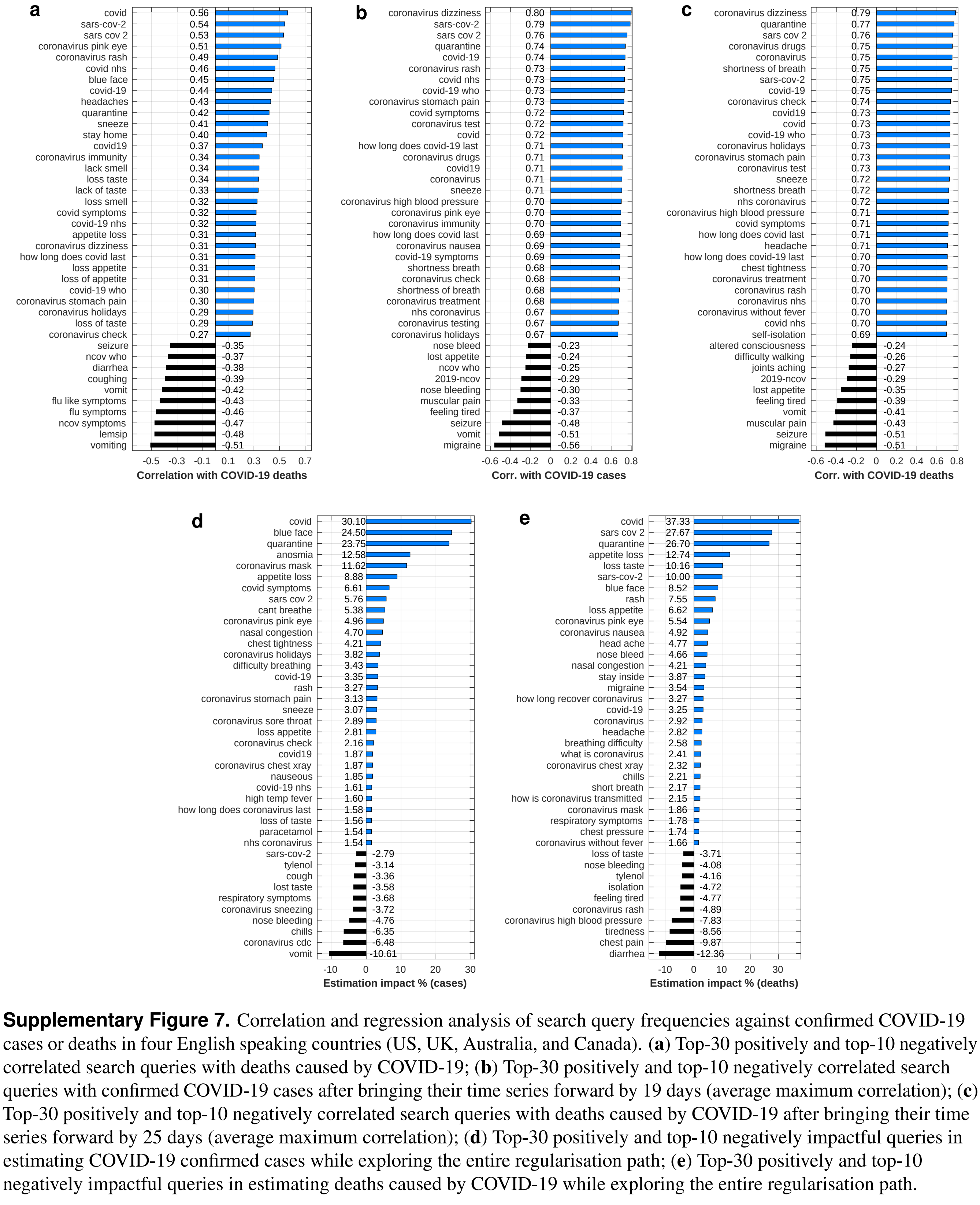

Supplementary Figure 7. Correlation and regression analysis of search query frequencies against confirmed COVID-19 cases or deaths in four English speaking countries (US, UK, Australia, and Canada). (**a**) Top-30 positively and top-10 negatively correlated search queries with deaths caused by COVID-19; (**b**) Top-30 positively and top-10 negatively correlated search queries with confirmed COVID-19 cases after bringing their time series forward by 19 days (average maximum correlation); (**c**) Top-30 positively and top-10 negatively correlated search queries with deaths caused by COVID-19 after bringing their time series forward by 25 days (average maximum correlation); (**d**) Top-30 positively and top-10 negatively impactful queries in estimating COVID-19 confirmed cases while exploring the entire regularisation path; (**e**) Top-30 positively and top-10 negatively impactful queries in estimating deaths caused by COVID-19 while exploring the entire regularisation path.

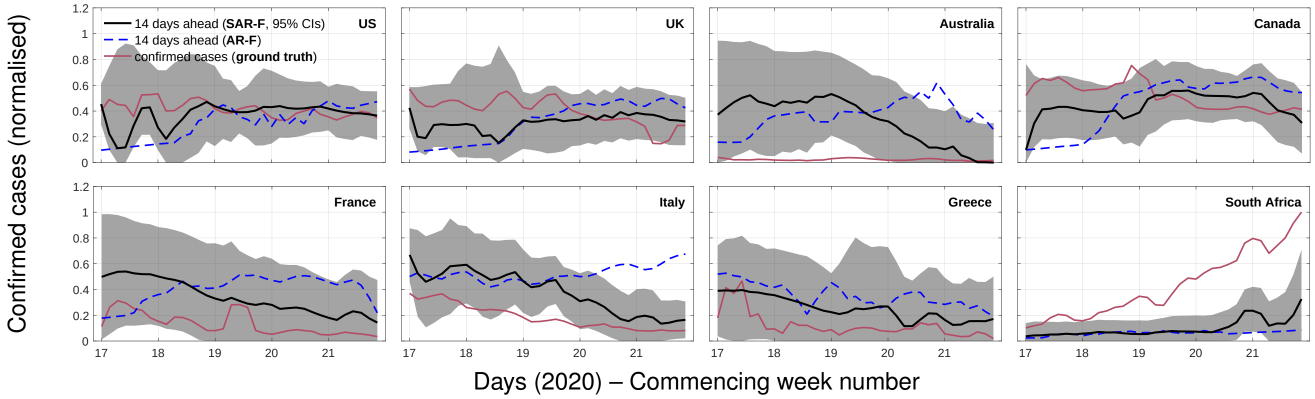

Supplementary Figure 8. 14-days ahead daily forecasting estimates of confirmed COVID-19 cases for 8 countries starting from April 20, 2020 or from the date by which a cumulative number of 250 cases have been reported (different date for each country). Deaths are depicted using a red line. The dashed blue line shows deaths forecasts from a strictly autoregressive model (**AR-F**). The black line shows deaths forecasts from a model that incorporates online search information (**SAR-F**). The shaded area denotes the corresponding 95% confidence intervals for the latter estimates. For a better visualisation, all values are normalised using min-max, and are smoothed using a 3-point moving average, centred around each day.

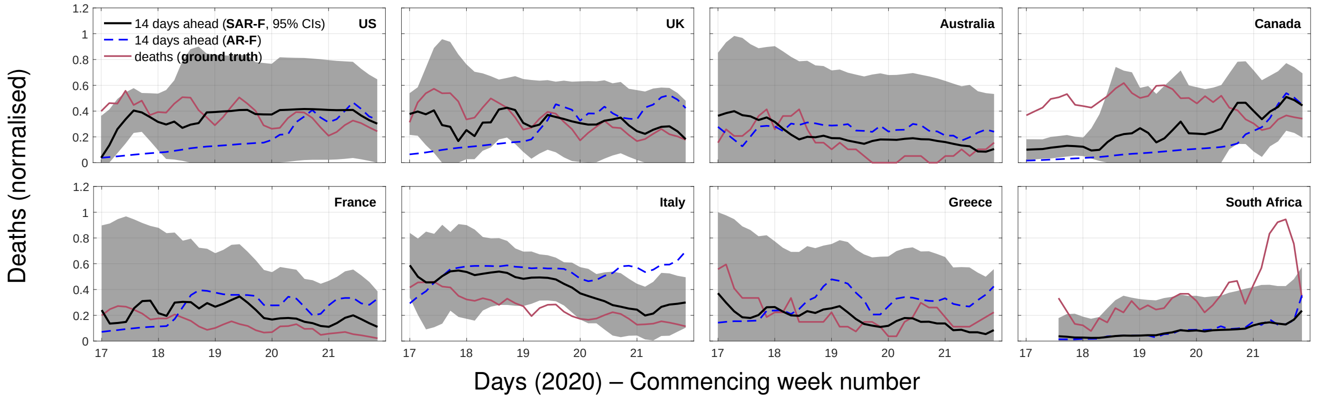

Supplementary Figure 9. 14-days ahead daily forecasting estimates of deaths caused by COVID-19 for 8 countries starting from April 20, 2020 or from the date by which a cumulative number of 10 deaths have been reported (differs for South Africa). Deaths are depicted using a red line. The dashed blue line shows deaths forecasts from a strictly autoregressive model (**AR-F**). The black line shows deaths forecasts from a model that incorporates online search information (**SAR-F**). The shaded area denotes the corresponding 95% confidence intervals for the latter estimates. For a better visualisation, all values are normalised using min-max, and are smoothed using a 3-point moving average, centred around each day.

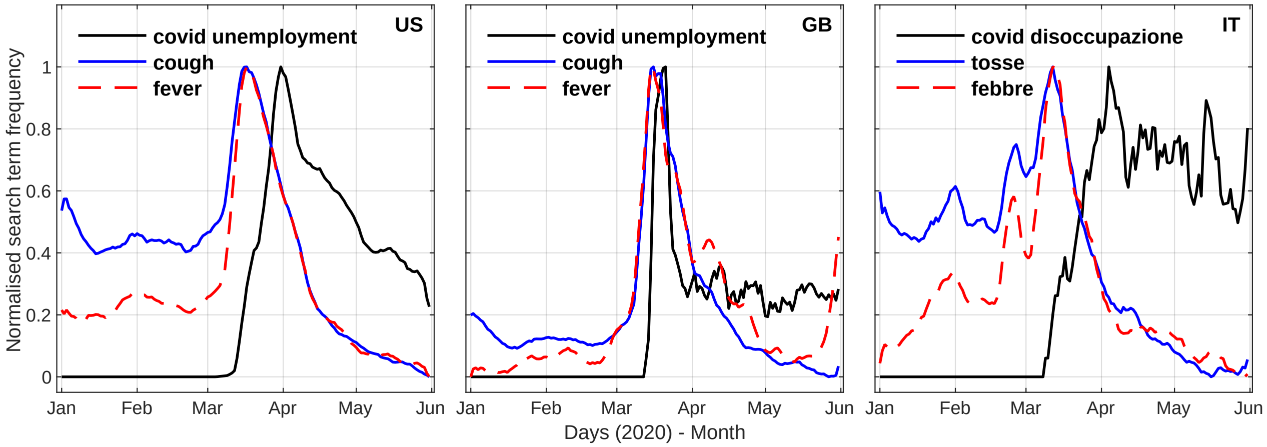

Supplementary Figure 10. Normalised (min-max) frequency time series for search queries that include the terms "covid unemployment" (black solid line), "cough" (blue solid line), or "fever" (red dashed line) across three countries, the US, UK, and Italy (translated in Italian language). Time series are smoothed using a 7-point moving average, centred around each day.

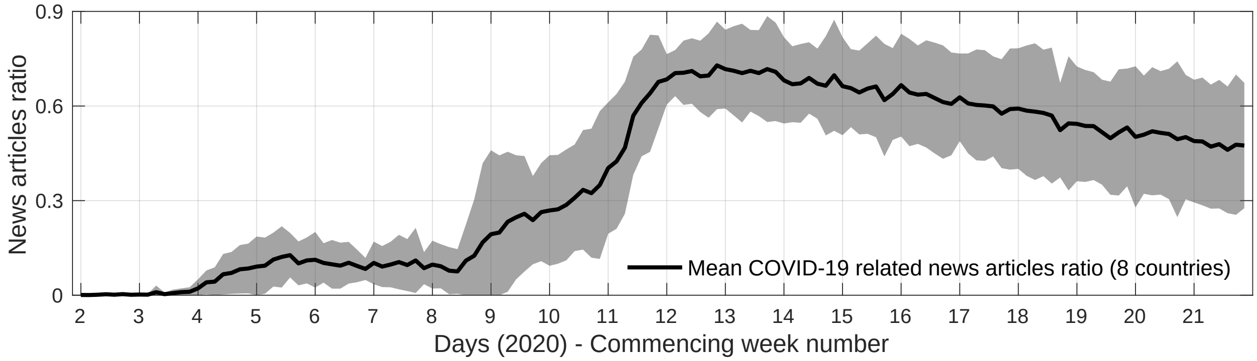

Supplementary Figure 11. Average daily news articles proportion about COVID-19 across all countries in our analysis and corresponding confidence intervals (two standard deviations above and below the mean).
